# Assessment of cognitive function, structural brain changes and fatigue 6 months after treatment of neuroborreliosis

**DOI:** 10.1007/s00415-022-11463-7

**Published:** 2022-11-15

**Authors:** Silje Andreassen, Elisabeth Margrete Stokke Lindland, Mona Kristiansen Beyer, Anne Marit Solheim, Unn Ljøstad, Åse Mygland, Åslaug Rudjord Lorentzen, Harald Reiso, Knut Jørgen Bjuland, Are Hugo Pripp, Hanne Flinstad Harbo, Gro Christine Christensen Løhaugen, Randi Eikeland

**Affiliations:** 1 Department of Pediatrics, Sørlandet Hospital Arendal, Postbox 416, 4604 Kristiansand, Norway; 2 Institute of Clinical Medicine, University of Oslo, Oslo, Norway; 3 Department of Radiology, Sørlandet Hospital, Arendal, Norway; 4 Division of Radiology and Nuclear Medicine, Oslo University Hospital, Oslo, Norway; 5 Department of Neurology, Sørlandet Hospital, Kristiansand, Norway; 6 Institute of Clinical Medicine, University of Bergen, Bergen, Norway; 7 Section of Habilitation, Sørlandet Hospital, Kristiansand, Norway; 8 Norwegian National Advisory Unit on Tick-Borne Diseases, Sørlandet Hospital, Kristiansand, Norway; 9 Oslo Centre of Biostatistics and Epidemiologi, Research Support Services, Oslo University Hospital, Oslo, Norway; 10 Faculty of Health Sciences, OsloMet–Oslo Metropolitan University, Oslo, Norway; 11 Department of Neurology, Oslo University Hospital, Oslo, Norway; 12 Department of Health and Nursing Sciences, University of Agder, Grimstad, Norway

**Keywords:** Lyme, Neuroborreliosis, Cognitive, Fatigue, MRI

## Abstract

**Background:**

Complete recovery after adequately treated neuroborreliosis is common, but studies report that some patients experience persistent symptoms like self-reported cognitive problems and fatigue. Persisting symptoms are often termed post-Lyme disease syndrome, of which etiology is not clearly understood. The aim of this study was to investigate cognitive function, possible structural changes in brain regions and level of fatigue. We have not found previous studies on neuroborreliosis that use standardized neuropsychological tests and MRI with advanced image processing to investigate if there are subtle regional changes in cortical thickness and brain volumes after treatment.

**Methods:**

We examined 68 patients treated for neuroborreliosis 6 months earlier and 66 healthy controls, with a comprehensive neuropsychological test protocol, quantitative structural MRI analysis of the brain and Fatigue Severity Scale.

**Results:**

We found no differences between the groups in either cognitive function, cortical thickness or brain volumes. The patients had higher score on Fatigue Severity Scale 3.8 vs. 2.9 (*p* = 0.001), and more patients (25.4%) than controls (5%) had severe fatigue (*p* = 0.002), but neither mean score nor proportion of patients with severe fatigue differed from findings in the general Norwegian population.

**Conclusion:**

The prognosis regarding cognitive function, brain MRI findings and fatigue after adequately treated neuroborreliosis is favorable.

## Introduction

Although neuroborreliosis is effectively treated with antibiotics, previous studies have found that 10–50% of the patients report persistent symptoms like fatigue, malaise, pain and subjective cognitive problems [1–5], a condition termed post-Lyme disease syndrome (PLDS) [6]. PLDS is a debated phenomenon and the etiology remains unclear, but among other hypotheses, systemic inflammation and immune responses leading to impaired central nervous system (CNS) function have been suggested [7–9]. Despite the high prevalence of self-reported cognitive problems, studies using cognitive tests show conflicting results. Some find reduced cognitive function, especially in verbal learning and memory [10–12], and processing speed [12, 13], while others find no differences between patients and healthy controls [14, 15]. Apart from leptomeningeal and cranial nerve enhancement in the acute phase, pathological findings on conventional MRI after treated neuroborreliosis are unusual [16–18]. MRI with high resolution and advanced image processing which allow quantification of cortical thickness and volume of brain regions has not been applied in patients with neuroborreliosis. The description of symptoms in PLDS resemble the description of myalgic encephalopathy (ME)/chronic fatigue syndrome, a condition also characterized by disabling fatigue, pain and malaise. Like PLDS, the etiology of ME remains unknown [19], and studies using quantitative MRI analysis on patients with chronic fatigue syndrome suggest alterations in grey and white matter [20], and reduced cortical volume and thickness in regions involved in attention, inhibition and memory retrieval [21].

The aim for this study was two-fold: firstly, we aimed to assess cognitive function with neuropsychological tests and measure self-reported fatigue in patients treated for neuroborreliosis six months previously as compared to a healthy age- and gender matched control group. Secondly, we aimed to assess cortical thickness and brain volumes in patients and controls using quantitative MRI.

## Materials and methods

### Recruitment and participants

**Fig. 1:**
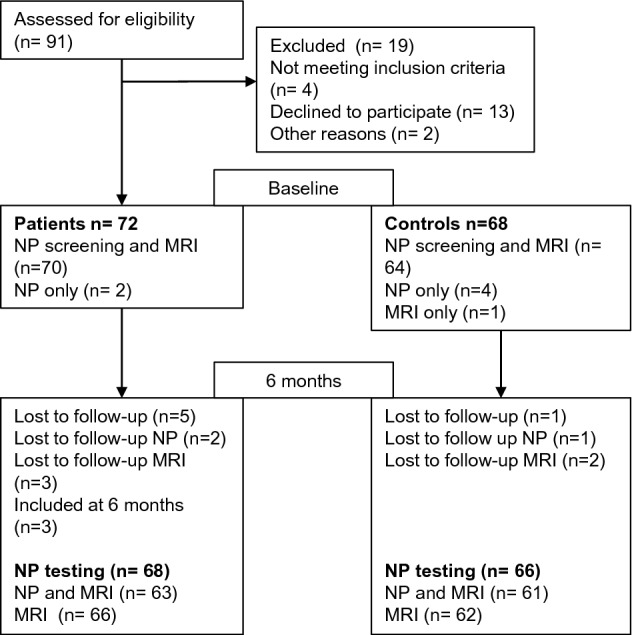
Flowchart of included patients and controls. *NP* neuropsychological

The study is part of a randomized, double-blinded non-inferiority trial comparing 2 and 6 weeks of doxycycline treatment in patients with acute neuroborreliosis [22]. Between November 2015 and December 2018, patients aged ≥ 18 years with possible or definite neuroborreliosis according to the European Federation of Neurological Societies criteria [23] were invited to participate. Healthy control persons matched by gender and age ± 2 years were included for comparison. In total, 72 patients and 68 controls participated in neuropsychological screening in the acute phase of neuroborreliosis, while 70 patients and 64 controls also underwent MRI scanning. Description of the subjects, the inclusion and exclusion criteria have been published previously [24]. In this follow-up, patients and controls underwent a comprehensive neuropsychological testing and brain MRI 6 months after inclusion. Patients prevented to participate in the acute phase were invited to participate later if they filled the inclusion criteria, and three patients were included at six-month follow-up (Fig. 1). In total, 68 patients and 66 control persons carried out neuropsychological assessment six months post-treatment. Sixty-six patients and 62 controls underwent brain MRI scanning, of which 63 patients and 61 controls carried out both neuropsychological testing and MRI.

### Neuropsychological assessment

Patients and controls underwent testing of general abilities using the subtests Block design, Matrixes, Vocabulary, Information, Digit-span, Arithmetic, Symbol search and Coding from Wechsler Adult Intelligence Scale 4th ed. to estimate full-scale IQ [25]. To assess different aspects of executive functions, we included Tower, Verbal fluency and Color word interference test from Delis Kaplan Executive Function System [26]. California Verbal Learning Test 2nd edition (CVLT-II) was used to assess verbal learning and memory [27], while visual memory was assessed by Family pictures from Wechsler Memory Scale 3^rd^ edition [28]. We defined a scaled score of two standard deviations (SD) below age-adjusted mean to be impaired. Patients who were also participating in the treatment trial (*n* = 58) were asked to grade subjective memory/concentration problems 6 months after treatment as no problems, mild without influence on daily living, or serious with influence on daily living.

### Assessment of fatigue

Fatigue severity scale (FSS) was used to identify level of fatigue [29]. FSS has been translated and validated in a Norwegian population, with an overall mean of 4 and a score of ≥ 5 considered to be a severe level of fatigue [30].

### MRI imaging

The whole brain scan for this study was a sagittal 3D T1 weighted MPRAGE (magnetization-prepared rapid gradient-echo) sequence. Subjects were included at two locations with different 3 T scanners, one with a Siemens Skyra and one with General Electric Signa where 55 patients and 58 control subjects were scanned on the Skyra and 11 patients and 5 controls were scanned on the Signa. Head coil with 64 and 32 channels, respectively, was used. The study protocol was set up to be as similar as possible with the following parameters (Skyra/Signa): Slice thickness 1.1/1.0 mm, field of view read 256 mm and phase 96.9%/256 mm, repetition time 2300 ms, echo time 2.98 ms/minimum and inversion time 900/943 ms.

### Image analysis

For the image analysis, we used FreeSurfer software package version 7.1.1 available at https://surfer.nmr.mgh.harvard.edu/. Details about the method are described elsewhere [31–34], briefly the cortical surface is automatically parcellated into 34 regions of interests (ROIs) in each hemisphere defined by FreeSurfer, and cortical thickness is calculated by measuring the distance between the grey/white matter boundary and the pial surface. ROIs were divided into left and right hemisphere lobes based on Desikan-Killiany atlas [35] resulting in following variables: right/left frontal lobe, right/left parietal lobe, right/left temporal lobe and right/left occipital lobe. Furthermore, we applied smoothing using a full-width-half-maximum (FWHM) to cortical thickness maps for each hemisphere with a Gaussian kernel of 30 mm. Cortical and cerebellar grey matter, cerebral and cerebellar white matter, subcortical volumes, corpus callosum and estimated intracranial volume (eICV) were obtained from automated procedures implemented in FreeSurfer. Subcortical structures in each hemisphere were combined resulting in the following dependent variables, hippocampus, amygdala, thalamus, caudate nucleus, putamen, globus pallidus and nucleus accumbens.

### Statistics

IBM SPSS statistics for Windows version 28 was used for statistical analysis [36]. Independent samples t-test was used to compare mean scores between the groups in neuropsychological tests and ROI based differences in cortical thickness in the brain lobes. Mann–Whitney U test was chosen to compare level of fatigue, as FSS was not normally distributed. For the surface-based analysis we fitted a general linear model at each vertex, with cortical thickness as dependent variable and age and gender as covariates to investigate group differences. We used general linear model with eICV as covariate to compare group differences in brain volumes between patients and controls.

We used Spearman rho to investigate correlation between FSS and cortical thickness, volumes and neuropsychological tests, and Chi-Square test to investigate proportions. To compensate for multiple comparisons, False Discovery Rate (FDR) was applied in the analysis of differences in mean neuropsychological tests scores, in ROI based cortical thickness and in brain volumes [37]. FDR was also used in vertex-wise comparison of cortical thickness. The only outcome measure on fatigue, FSS was not corrected. Additional outcome measures, proportions and associations were not corrected.

### Missing data

Seven patients were unable to perform one or more of the neuropsychological tests, and five patients missed one or more subtests at random. For the control group, one participant was unable to perform two neuropsychological tests, while four participants missed one or more subtests at random. Missing data were handled by pairwise deletion in the analysis.

For the FreeSurfer analysis, one patient and five controls were excluded from the analysis due to errors in segmentation.

## Results

Demographic and clinical data for patients and controls are presented in Table 1. Mean neuropsychological test results, including full scale IQ and scores on FSS are presented in Table 2. We found no differences in mean scores between the groups in any of the neuropsychological tests, but a larger proportion (21.7%) of the patients had very low scores (-2 SD) on the first trial in CVLT-II compared to controls (6.2%), (*p* = 0.011). There were no differences in proportions in any other subtests. The patients had a higher level of fatigue. Out of 63 patients, 16 (25.4%) reported severe fatigue ≥ 5, vs. 3 (5.0%) in the control group (*p* = 0.002). Out of 58 patients being asked, 41 (70.7%) reported no subjective memory/concentration problems, while 17 (29.3%) reported mild problems without influence of daily living. No patients reported serious problems with influence of daily living. Fifty-four of these patients also completed CVLT-II. Out of 38 patients reporting no subjective memory/concentration problems, 10 patients (26.3%) had very low score on CVLT-II trial 1, while 28 patients (73.7%) had normal scores. Out of 16 patients reporting mild problems, 3 (18.8%) had very low score on trial 1, while 13 (81.3%) were in the normal range. No association was found between subjective memory/ concentration problems and very low score on CVLT-II trial 1 (*p* = 0.553, unadjusted). Patients who were on sick-leave related to neuroborreliosis (*n* = 11), did not have lower scores on neuropsychological tests compared with patients not on sick-leave, but a higher level of fatigue (FSS 5.4 vs. 3.5, *p* = 0.002, unadjusted).

**Table 1:** Characteristics of patients and controls

	Patients (*n* = 68)	Controls (*n* = 66)	*p* value
Male/female	35/33	32/34	0.861
Age in years (range)	58 (27–82)	58 (26–81)	0.659
Mean SES	3.5	3.6	0.849
Full scale IQ (SD)	101.4 (13.4)	102.2 (12.5)	0.690
Work status 6 months post treatment			0.140
Full time/part time job	26	36	
Age pension	23	22	
Disability pension	6	4	
Sick leave full time (related to NB)	4 (4)	1	
Sick leave part time (related to NB)	8 (7)	1	
Other	1	2	
Definite NB	55	n/a	
Possible NB	13	n/a	

Level of significance *p* < 0.05

*SES* socioeconomic status, *IQ* intelligence quotient, *NB* neuroborreliosis

**Table 2:** Mean neuropsychological test results and level of fatigue

Test	Patients (*n* = 68)	Controls (*n* = 66)	*p* value unadjusted	*p* value adjusted
	Mean (SD)	Mean (SD)		
General abilities				
Estimated full scale IQ	101.4 (13.4)	102.2 (12.5)	0.690	0.833
Executive functions				
Letter fluency raw	40.3 (13.8)	41.4 (11.8)	0.620	0.833
Category fluency raw	46.1 (10.2)	46.0 (10.4)	0.980	0.980
Category switching raw	13.4 (2.8)	13.9 (2.7)	0.288	0.480
CW naming raw score	31.6 (6.7)	31.5 (5.1)	0.919	0.980
CW read raw	22.9 (4.3)	22.2 (3.5)	0.279	0.480
CW inhibition raw score	61.8 (20)	57.0 (14.5)	0.111	0.405
CW switching raw score	70.1 (22.1)	61.3 (15.7)	0.007*	0.053
Tower total raw	17.2 (3.9)	17.9 (3.5)	0.257	0.480
Visual memory				
Family immediate raw	31.6 (9.3)	31.0 (10.3)	0.722	0.833
Family delayed raw	30.4 (10.0)	31.3 (9.8)	0.586	0.833
Verbal memory				
Trial 1 raw	4.6 (1.6)	5.3 (1.4)	0.007*	0.053
Trial 1–5 raw	42.6 (9.9)	46.3 (9.3)	0.032*	0.160
Short delay free raw	9.1 (3.2)	9.9 (3.0)	0.135	0.405
Long delay free raw	9.7 (3.2)	10.5 (3.4)	0.189	0.473
Fatigue				
FSS	3.8 (1.7)	2.9 (1.3)	0.001*	

Adjusted *p* values are corrected for multiple testing using False Discovery Rate (FDR). Level of significance, *p* < 0.05

*IQ* intelligence quotient, *CW* color word, *FSS* Fatigue Severity Scale

**Fig. 2:**
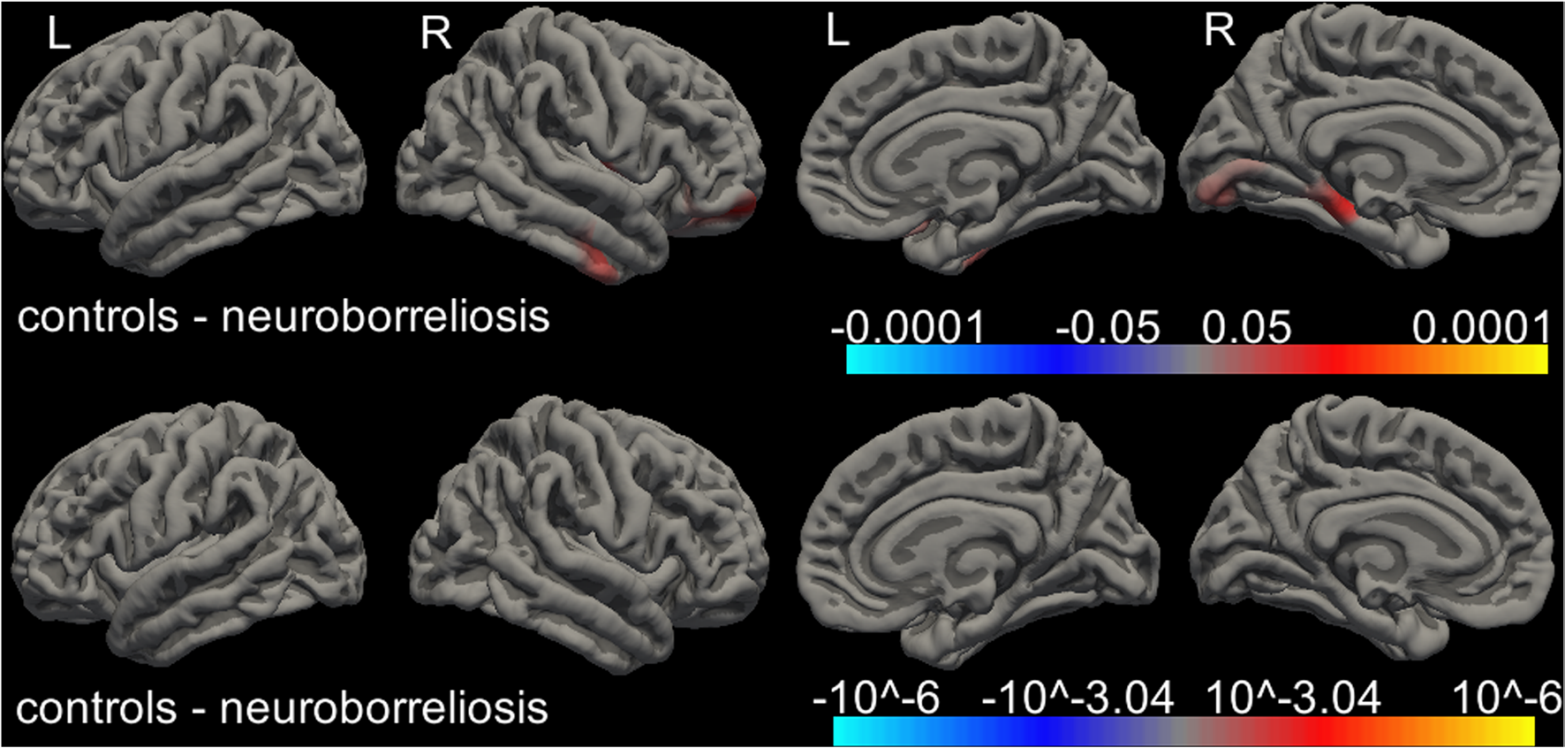
Cortical thickness maps of 65 patients and 57 controls without (upper panel) and with (lower panel) false discovery rate (FDR) correction. The brain areas with significant differences are shown in color, and the color scale shows dynamic range of the statistical change in *p* values. Red represents thinner cortex in patients compared with controls, but was not significant after FDR correction. *R* right, *L* left

We found no differences in cortical thickness in frontal, parietal, temporal or occipital lobe (Table 3). Whole brain vertex-wise comparison revealed no differences in cortical thickness between groups after correcting for multiple comparison (Fig. 2). Moreover, we found no differences in brain volumes between the groups (Table 4).

**Table 3:** Mean thickness of lobes in right and left hemispheres

Cortical thickness	Patients, *n* = 65	Controls, *n* = 57	*p* value unadjusted	*p* value adjusted
	Mean mm (SD)	Mean mm (SD)		
RH frontal	2.41 (0.08)	2.42 (0.08)	0.877	0.877
LH frontal	2.44 (0.08)	2.47 (0.08)	0.029*	0.152
RH temporal	2.79 (0.14)	2.83 (0.09)	0.038*	0.152
LH temporal	2.74 (0.17)	2.79 (0.11)	0.068	0.181
RH parietal	2.30 (0.09)	2.31 (0.10)	0.320	0.507
LH parietal	2.27 (0.09)	2.31 (0.09)	0.252	0.504
RH occipital	1.94 (0.12)	1.96 (0.12)	0.380	0.507
LH occipital	1.91 (0.11)	1.92 (0.11)	0.639	0.730

Corrected for multiple testing using False Discovery Rate (FDR). Level of significance *p* < 0.05

*LH* left hemisphere, *RH* right hemisphere

**Table 4:** Comparison of brain volumes controlled for intracranial volume in patients treated for neuroborreliosis and healthy controls

Brain volumes (in mL)	Patients (*n* = 65)		Controls (*n* = 57)		*p* value unadjusted	*p* value adjusted
	Adjusted mean	(95% CI)	Adjusted mean	(95% CI)		
Cortex volume	470.41	(461.75–479.07)	480.28	(471.03–489.53)	0.128	0.472
Cerebral white matter	463.08	(453.11–473.05)	464.14	(453.52–474.83)	0.883	0.922
Hippocampus	3.97	(3.86–4.08)	4.07	(3.95–4.18)	0.236	0.472
Amygdala	1.70	(1.65–1.76)	1.75	(1.70–1.81)	0.202	0.472
Thalamus	7.00	(6.82–7.18)	7.05	(6.86–7.24)	0.730	0.922
Caudate nucleus	3.62	(3.53–3.71)	3.59	(3.49–3.68)	0.579	0.472
Putamen	4.96	(4.83–5.09)	4.95	(4.81–5.09)	0.912	0.922
Globus pallidus	1.96	(1.91–2.01)	1.92	(1.87–1.97)	0.227	0.472
Nucleus accumbens	0.58	(0.55–0.61)	0.59	(0.56–0.62)	0.829	0.922
Cerebellar grey matter	53.56	(52.32–54.79)	51.91	(50.57–53.22)	0.074	0.472
Cerebellar white matter	15.44	(15.03–15.85)	14.90	(14.46–15.34)	0.079	0.472
Corpus callosum	0.68	(0.65–0.70)	0.68	(0.65–0.70)	0.922	0.922

Adjusted mean: estimated marginal mean from a general linear model with group as fixed factor and total intracranial volume as covariate

Corrected for multiple testing using False Discovery Rate (FDR). Level of significance, *p* < 0.05

There was no correlation between FSS and cortical thickness in brain lobes and brain volumes. In the patient group, we found a weak, but significant correlation between FSS and color word naming (*r* = 0.329, *p* = 0.009), color word inhibition (*r* = 0.334, *p* = 0.008) and color word inhibition switching (*r* = 0.332, *p* = 0.008). *P*-values in analysis of correlations are unadjusted.

## Discussion

In this prospective cohort of 68 well-characterized patients with adequately treated neuroborreliosis, we found no reduction in cognitive functions measured with neuropsychological tests, and no alterations in cortical thickness and brain volumes compared with a healthy control group. The only variable that remained significant after correcting for multiple comparisons was a higher level of self-reported fatigue in the patient group. Although some studies find self-reported cognitive problems in a considerable proportion of patients with treated neuroborreliosis [2, 4], we were not able to find reduced cognitive function in our cohort. Our results are in line with Dersch et al. [15] and Berende et al. [14], but contrary to studies finding reduced verbal learning/memory and attention/executive function [10, 11, 38–40]. There are several possible explanations for the different results. We found reduced scores in some subtests on CVLT-II and Color Word, resembling the findings by Eikeland et al. [10] who examined a cohort of neuroborreliosis patients 30 months after treatment. Unlike Eikeland et al., however, we corrected for multiple comparisons due to the high number of outcome measures, and the difference did not survive correction. Both the study by Benke et al. [38] and Touradji et al. [11] were retrospective studies, and could be hampered by sampling bias. Furthermore, the latter one did not include a healthy control group, but instead relied upon normative data. Keilp et al. [41] included only patients who had persistent health problems defined as PLDS, making it a selected group in that respect. A proportion of the patients in our cohort had very low scores on the first trial in CVLT-II, but not in total learning, immediate or delayed memory. To claim that a proportion of patients have severe learning and memory problems might be an overinterpretation of such a single finding, especially as 70% of the patients being asked, reported no problems with memory or concentration, while the rest reported only mild problems without influence in daily living. Furthermore, the majority of patients reporting mild problems (81.3%) had scores within normal range. The item subjective concentration/ memory problem is part of a clinical composite score that is unvalidated, but has been used in previous clinical studies [42, 43]. Since none of the controls were asked to grade subjective memory or concentration problems, and the clinical composite score is unvalidated, we do not know whether mild subjective concentration/ memory problems are frequent in the control group or the general population. A modest association between self-reported cognitive problems and results on neuropsychological test-results has been documented previously, both in patients with neuroborreliosis [14] and other patient groups [44, 45]. We did not include a questionnaire to assess patients’ subjective cognitive problems in detail, nor did we ask them about their expectancy regarding the outcome when included in the acute phase. A recent study on expectancy regarding outcome showed that both premorbid function and positive expectancy were related to a more beneficial outcome in patients [46].

Patients reported higher level of fatigue compared with the controls, and patients on sick leave related to neuroborreliosis reported higher level of fatigue compared with patients not on sick leave. Mean FSS score was 3.8 in the overall patient group and 2.9 in the control group and 25.4% of the patients versus 5% of the controls reported severe fatigue defined as FSS ≥ 5. Interestingly though, the mean level of fatigue among patients and the proportion of patients who reported severe fatigue, was comparable with findings in the general Norwegian population (mean 3.8 vs 4.0 and 25.4% vs 23.1%, respectively) [30]. A possible explanation for the difference between patients and controls could be that control persons who participate in a research project with a comprehensive protocol might have lower fatigue in the first place. We found a low, but significant negative correlation with fatigue and tests assessing processing speed, inhibition and mental flexibility in the patient group, but not in the control group. In this analysis, we did not correct for multiple comparison, but the association is plausible given these subtests are demanding and performance might be influenced by fatigue.

We did not find any differences in cortical thickness or volumes between patients and controls. Research in patients with ME have shown these patients tend to score lower in certain cognitive domains, mainly reduced processing speed [47]. Imaging studies in patients with ME have shown alterations in cortical and subcortical volumes, both positive and negative findings due to methodological variations [48]. A recent MRI study on cortical thickness and brain volumes found reduced cortical volumes and thickness in patients with ME compared with a healthy control group, and reductions correlated with fatigue [21]. Unlike these studies, we were not able to detect any differences between patients and controls neither in an extensive neuropsychological assessment covering different cognitive domains, nor in cortical thickness and brain volumes. Moreover, we found no association between level of fatigue and cortical thickness and volumes. Contrary to the patients in the latter study, our patients had higher fatigue scores compared to our control group, but not compared to the general Norwegian population. Since we found no differences in cognitive function between the groups, and only a moderate level of fatigue in the patient group, absence of alterations in brain structures may not be surprising.

The strengths of our study are a large cohort of patients with well-characterized neuroborreliosis and an age and gender matched control group. We included a comprehensive neuropsychological protocol covering several cognitive domains, including full-scale IQ to ensure premorbid cognitive function in the patient group was comparable with the control group. Seven patients missed one or more subtests due to fatigue, while only one of the control persons missed one or more subtests for the same reason. We cannot rule out missing data may have influenced the results on some of the neuropsychological tests. None of the participants missed Color Word subtests due to fatigue. As these subtests were the only ones that weakly correlated with fatigue, we interpret the influence of missing data to be modest. Considering no other studies have investigated morphological alterations in patients with neuroborreliosis using the same method, our research questions regarding MRI findings of cortical thickness, volume and subcortical volume were wide and explorative. We chose to merge some regions of interest and volumes instead of focusing on very numerous specific regions, and minor changes in specific structures might have been wiped-out.

## Conclusion

Cognitive function, cortical thickness, and brain volumes do not seem to be affected in patients with adequately treated neuroborreliosis. The patients report a higher level of fatigue compared to the control group, but not compared to the general Norwegian population. Overall, our results indicate a favorable outcome in a majority of patients with adequately treated neuroborreliosis.

## Data Availability

Data are available on reasonable request.
